# Non-contrast-enhanced MR-angiography of Extracranial Arteries in Acute Ischemic Stroke at 1.5 Tesla Using Relaxation-Enhanced Angiography Without Contrast and Triggering (REACT)

**DOI:** 10.1007/s00062-024-01458-4

**Published:** 2024-09-24

**Authors:** Jan P. Janssen, Sarah Rose, Kenan Kaya, Robert Terzis, Robert Hahnfeldt, Roman J. Gertz, Lukas Goertz, Andra-Iza Iuga, Jan-Peter Grunz, Christoph Kabbasch, Philip Rauen, Thorsten Persigehl, Kilian Weiss, Jan Borggrefe, Lenhard Pennig, Carsten Gietzen

**Affiliations:** 1 Faculty of Medicine and University Hospital Cologne, University of Cologne, Institute for Diagnostic and Interventional Radiology, Kerpener Straße 62, 50937 Cologne, Germany; 2 University Hospital Wuerzburg, Institute for Diagnostic and Interventional Radiology, Wuerzburg, Germany; 3 University of Wisconsin-Madison, Madison, WI USA; 4 Philips GmbH, Hamburg, Germany; 5 Department of Radiology, Neuroradiology and Nuclear Medicine, Johannes Wesling University Hospital, Ruhr University Bochum, Bochum, Germany

**Keywords:** Magnetic resonance angiography, Stroke, Non-contrast-enhanced magnetic resonance angiography, ICA stenosis, Carotid arteries

## Abstract

**Purpose:**

To evaluate a novel flow-independent sequence (Relaxation-Enhanced Angiography without Contrast and Triggering (REACT)) for imaging of the extracranial arteries in acute ischemic stroke (AIS) at 1.5 T.

**Methods:**

This retrospective single-center study included 47 AIS patients who received REACT (scan time: 3:01 min) and contrast-enhanced MRA (CE-MRA) of the extracranial arteries at 1.5 T in clinical routine. Two radiologists assessed scans for proximal internal carotid artery (ICA) stenosis, stated their diagnostic confidence and rated the image quality of cervical arteries, impact of artifacts and image noise. Apparent signal- and contrast-to-noise ratios (aSNR/aCNR) were measured for the common carotid artery and ICA.

**Results:**

REACT achieved a sensitivity of 95.0% and a specificity of 97.3% for ICA stenoses in high agreement with CE-MRA (κ = 0.83) with equal diagnostic confidence (*p* = 0.22). Image quality was rated higher for CE-MRA at the aortic arch (*p* = 0.002) and vertebral arteries (*p* < 0.001), whereas REACT provided superior results for the extracranial ICA (*p* = 0.008). Both sequences were only slightly affected by artifacts (*p* = 0.60), while image noise was more pronounced in CE-MRA (*p* < 0.001) in line with higher aSNR (*p* < 0.001) and aCNR (*p* < 0.001) values in REACT for all vessels.

**Conclusion:**

Given its good diagnostic performance while yielding comparable image quality and scan time to CE-MRA, REACT may be suitable for the imaging of the extracranial arteries in acute ischemic stroke at 1.5 T.

**Supplementary Information:**

The online version of this article (10.1007/s00062-024-01458-4) contains supplementary material, which is available to authorized users.

## Introduction

In acute ischemic stroke (AIS), accurate depiction of the extracranial arteries is of great importance in order to detect potential causes like internal carotid artery (ICA) stenosis or occlusion [1]. Treatment options such as endarterectomy or stent implantation can significantly reduce the risk of further strokes and improve patient outcomes [2]. Digital subtraction angiography (DSA) is the gold standard for imaging of the cervical arteries due to its high diagnostic accuracy and potential treatment options. Nevertheless, it is not regarded as the first imaging modality of choice given its invasiveness with potential associated complications and high costs [3]. In the setting of severe AIS with clear onset, CT including CT angiography (CTA) of the head and neck is considered as a fast, non-invasive imaging modality of choice [4]. It is widely available, highly reliable, and provides sufficient information for relevant treatment decisions [4, 5]. Compared to DSA, CTA provides a sensitivity of 75–97% and specificity of 82–99% for relevant (≥ 50%) ICA stenosis and provides information on plaque composition [6–9]. However, stenoses with highly calcified plaques may be overestimated [10]. Additional disadvantages include radiation exposure and the use of iodine-based contrast agents, which can lead to nephropathy or thyrotoxicosis [11, 12]. Albeit not suitable for evaluating the extent of stroke, duplex ultrasound (DUS) is a widely available, radiation- and contrast-free alternative for the evaluation of carotid arteries, yielding a sensitivity of 85–97% and specificity of 70–99% for relevant ICA stenosis compared to DSA [13] while enabling assessment of plaque composition [14]. However, this method has some disadvantages, including dependence on the physician’s experience and morphological issues such as obesity and vessel elongation.

MRI represents a valuable alternative for less affected patients or those with unclear onset [4, 15]. Contrast-enhanced MRA (CE-MRA) provides a radiation-free depiction of the cervical arteries with a sensitivity of 66–97% and specificity of 91–96% for relevant ICA stenosis and plaque assessment, potentially supplemented by additional dedicated sequences [9, 16–18]. However, performance in low and middle grade stenosis is less evident and limitations associated with gadolinium-based contrast agents remain, e.g., allergic reactions, potential risk of nephrogenic systemic fibrosis in end-stage renal disease, mistiming of image acquisition, and unknown long-term effects of gadolinium deposition in the body [19, 20].

Consequently, continuous efforts are made to develop non-CE-MRA sequences that provide reliable visualization of vascular pathologies. While time-of-flight (TOF)-MRA has become the clinical standard for intracranial, horizontally aligned vessels, this technique is regarded as unsuitable for vertically aligned arteries of the neck due to long acquisition times and overestimation of ICA stenosis given its flow-dependency [21, 22]. Recently, two promising sequences, Quiescent Interval Slice Selective (QISS)-MRA [23] and Relaxation-Enhanced Angiography without Contrast and Triggering (REACT) [24], have been introduced. QISS-MRA is a 2D flow-dependent technique employing dedicated radiofrequency pulses to suppress background and venous signals followed by a fast low angle shot (FLASH) readout [25, 26]. REACT uses an inversion recovery (IR) prepulse and T2 preparation with Dixon readout for flow-independent 3D isotropic non-CE-MRA [24]. Both REACT and QISS-MRA have shown encouraging results for the imaging of the supraaortal arteries at 3 T [25–29]. Furthermore QISS-MRA has also been introduced to carotid MRA at 1.5 T, showing comparable image quality and diagnostic accuracy to CE-MRA for the detection of ICA stenosis [30]. While the REACT sequence has shown promising results in the imaging of the pulmonary vasculature [31, 32] and the thoracic aorta [33, 34] at 1.5 T, its performance regarding the depiction of the neck arteries at 1.5 T is unknown, potentially limiting the use of REACT with respect to carotid arteries in elderly patients, who may yield relative contraindications to 3 T MRI due to implants.

The aim of this study was to evaluate the REACT sequence as an alternative to CE-MRA for imaging of the extracranial arteries using a large cohort of patients with AIS which received neck MRA at 1.5 T by comparing the subjective and objective assessment of image quality and ICA stenosis.

## Materials and Methods

Ethical approval was waived by the local institutional review board in view of the retrospective nature of the study and all the procedures being performed were part of the routine care.

### Patient Population

The image database of a tertiary care university hospital was screened for stroke MRIs at 1.5 T between August 2020 and May 2023. In accordance with the established in-house standards, patients exhibiting a National Institutes of Health Stroke Scale (NIHSS) [35] upon admission of less than 6 and a documented stroke onset of more than 4.5 h, or an indeterminate time frame, were referred for MRI. Patients who received additional MRA of the neck region including both CE-MRA and REACT were included in the study. Patients with incomplete scan data, severe artifacts or technical failure were excluded.

Age, gender, cardiovascular risk factors, NIHSS at admission, the results of supplementary DUS, and modified Rankin Scale (mRS) [4] at discharge were retrieved from the medical records of the hospital database.

### MRI

A commercially available 1.5 T MRI system (Philips Ingenia, *Philips Healthcare, Best, The Netherlands*) with a 20-channel coil for head and neck was used for all examinations. The AIS protocol included diffusion-weighted imaging, fluid-attenuated inversion recovery sequences, axial susceptibility-weighted imaging and an intracranial 3D TOF-MRA sequence. In addition, all patients received the REACT sequence (index test) and a CE-MRA (reference standard) of the neck.

REACT is a non-triggered, 3D isotropic flow-independent sequence based on T2 preparation and inversion recovery pulses (allowing the enhancement of the native blood signal) followed by a 3D Dixon readout (mDIXON XD, *Philips Healthcare, Best, The Netherlands*) to suppress the background signal [24]. Data acquisition was performed in the coronal plane, water-only as well as in- and out-of-phase images were reconstructed [36].

For CE-MRA, a 3D spoiled gradient-echo T1 sequence was used. An unenhanced study for subtraction of the background was acquired before administration of the contrast agent. Gadolinium-based contrast agent (Clariscan, *GE Healthcare, Chicago, IL, USA; *0.2 mL/kg body weight) was injected via an antecubital vein at a flow rate of 2 mL/s followed by a 30 mL saline flush. Non-triggered data acquisition in the coronary plane was initiated as soon as the bolus tracking sequence registered the arrival of the contrast agent in the aortic arch.

The acquisition of both sequences was accelerated using Compressed SENSE (*Philips Healthcare, Best, The Netherlands*), a technique based on parallel imaging and compressed sensing [37]. An acceleration factor of 4 was used for REACT (scan time 3:01 min), while CE-MRA was accelerated by a factor of 5 (scan time: 3:10 min).

Table 1 shows an overview of the scan parameters of the applied extracranial MRA sequences.

**Table 1:** Scan parameters of the MRA sequences

	CE-MRA	REACT
K‑space trajectory	Cartesian	Cartesian
Acquisition orientation	Coronal	Coronal
Acquired voxel size	0.7 × 0.7 × 0.7 mm^3^	1.5 × 1.5 × 1.5 mm^3^
Reconstructed voxel size	0.5 × 0.5 × 0.5 mm^3^	0.69 × 0.69 × 0.75 mm^3^
Field of view (FHxRLxAP)	320 × 320 × 90 mm^3^	300 × 300 × 80 mm^3^
T2 preparation	n/a	50 ms, refocusing pulses: 4
Repetition time	6.9 ms	7 ms
Echo time (1/2)	1.96 ms	1.93/4.5 ms
Flip angle	35°	12°
Temporal resolution	1 s	n/a
Compressed SENSE factor	5	4
Subtraction	CE-native	–
Scan time	03:10 min	03:01 min

*CE* contrast-enhanced, *REACT* Relaxation-Enhanced Angiography without Contrast and Triggering, *FH* feet head, *RL* right left, *AP* anterior posterior

### Image Analysis

Image analysis was performed with a commercially available image viewer (DeepUnity Diagnost, release 1.1.1.1, *Dedalus Healthcare Systems Group, Bonn, Germany*). For assessment of ICA stenosis and evaluation of subjective image quality, a radiologist in training with two years (R1) and a board-certified radiologist with six years (R2) of experience in MRA assessed the images independently in separate sessions and in random order. Readers were blinded to clinical and patient data. Prior to evaluation, both readers were instructed by a board-certified neuroradiologist (18 years of experience in MRA) regarding the sufficient evaluation of image data using exemplary images of different grades of ICA stenosis and distinct levels of image quality of REACT and CE-MRA.

Additionally, a time interval of one month was set between evaluation of REACT and CE-MRA datasets to reduce recall bias. Evaluation of objective image quality was performed by a doctoral candidate (R3) and verified by the candidate’s academic supervisor, a board-certified radiologist with seven years of experience in MRA.

#### ICA Stenosis Assessment

Based on the North American Symptomatic Carotid Endarterectomy Trial criteria [38], ICA stenoses were divided into 5 grades (1: no stenosis; 2: < 50% stenosis; 3: 50–69% stenosis; 4: 70–99%; 5: total occlusion). When numerous stenoses were present, the most stenotic lesion was used for further assessment. A stenosis grade ≥ 50% was interpreted as clinically relevant. The diagnostic confidence of stenosis grading was rated on a 3-point scale for each side and patient (1: poor; 2: moderate; 3: good).

#### Subjective Image Quality

The following vessel sections were evaluated in terms of subjective image quality in the same sessions: Aortic arch and its adjacent branches; Bilateral common carotid artery (CCA), ICA (extracranial and petrous segment), vertebral artery (segments V1–V3). All segments were evaluated using a 5-point scale for the following categories:

1. General impression of image quality: 1 = non-diagnostic (image quality being insufficient for diagnosis); 2 = poor (image quality leading to impaired diagnosis); 3 = moderate (image quality being acceptable for diagnosis); 4 = good (image quality leading to confident diagnosis); 5 = excellent (image quality providing highly confident diagnosis).
2. Image noise: 1 = non-diagnostic (very high noise with images being insufficient for diagnosis); 2 = high image noise (high noise leading to impaired diagnosis); 3 = moderate image noise (mediocre noise leading to images being acceptable for diagnosis); 4 = low image noise (low effect on images); 5 = image noise with no effect on image quality.
3. Overall presence of artifacts (e.g., blurring, banding, pulsation, and parallel imaging reconstruction artifacts): 1 = non-diagnostic (strong artifacts leading to a non-diagnostic study); 2 = severe artifacts (artifacts with high effect on image quality); 3 = moderate artifacts (artifacts with moderate effect on artifacts); 4 = minor artifacts (artifacts with low effect on artifacts); 5 = no artifacts.

#### Fat-water Swapping Artifacts

A radiologist with three years of experience in MRA (R4) evaluated water maps of REACT for the presence of fat-water swapping artifacts, as well as the corresponding signal of the in-phase image at the respective signal loss of the water map if present [28, 29, 39].

#### Objective Image Quality

Apparent signal-to-noise ratio (aSNR) and apparent contrast-to-noise ratio (aCNR) were assessed by placing ROIs in both REACT (water-only) and CE-MRA (subtracted images) source images. Signal intensity of the vessels was measured 3 cm proximal (CCA) and 3 cm distal (ICA) to the carotid bifurcation on both sides. Additionally, a ROI was placed at each measurement level in the adjacent sternocleidomastoid muscle to determine the background noise.

The following formulas were used to calculate aSNR and aCNR [28]:

$$aSNR=\frac{SI_{v}}{\sigma _{{SI_{m}}}}\qquad aCNR=\frac{\left(SI_{v}-SI_{m}\right)}{\sigma _{{SI_{m}}}}$$

Hereby, *SI*_*v*_ is the signal intensity of the measured vessel, *SI*_*m*_ is the signal intensity of the adjacent muscle and $\sigma _{{SI_{m}}}$ being the corresponding standard deviation of the signal intensity in the muscle.

Mean values of aCNR and aSNR for both sides and each level were calculated and used for further analysis.

### Statistical Analysis

Statistical analysis was performed using the R programming language v. 4.0.2 and the open-source software RStudio (posit.co, accessed on 12 January 2024). Categorical variables are presented as frequencies and corresponding percentages. The Shapiro-Wilk test was applied to test for normal distribution (ND). Quantitative data are indicated as the mean ± standard deviation (if ND) or as median with IQR (if not ND). Subjective ratings are presented as frequencies and corresponding percentages and median with interquartile range. Differences were compared with Student’s *t* test (ND), or Wilcoxon signed rank test (if not ND) and calculated by averaging the measurements of all readers.

Kendall’s coefficient of concordance (*W*) was calculated to assess interobserver agreement for subjective image quality. Cohen’s kappa (κ) was used to evaluate the intersequence agreement between REACT and CE-MRA as well as interobserver agreement regarding ICA stenosis grading in REACT (0.01–0.2 slight, 0.21–0.4 fair, 0.41–0.6 moderate, 0.61–0.8 substantial, and 0.81–0.99 almost perfect). Sensitivity and specificity of REACT were calculated pooled for both readers using CE-MRA as the reference standard. In addition, the sensitivity and specificity of REACT and CE-MRA (pooled for both readers) were calculated using the DUS results from the medical records.

For all tests, a two-tailed *p* value of < 0.05 was considered statistically significant.

## Results

### Study Population and Baseline Characteristics

**Fig. 1:**
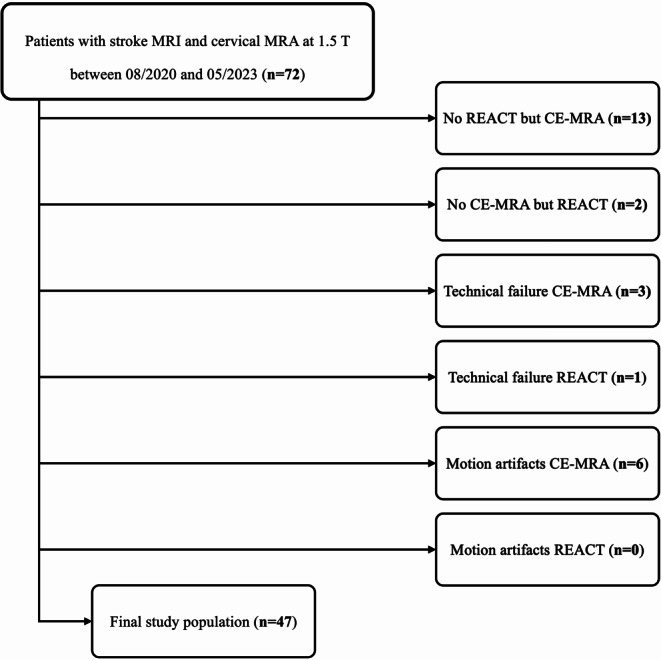
Workflow for inclusion and exclusion of patients. (*REACT* Relaxation-Enhanced Angiography without Contrast and Triggering, *CE-MRA* contrast-enhanced magnetic resonance angiography)

Seventy-two patients with AIS who received a stroke protocol at 1.5 T with MRA of the extracranial arteries were identified. Twenty-five patients were excluded due to either a missing sequence, technical failure, or severe artifacts such as motion. The workflow for inclusion and exclusion of study participants is shown in Fig. 1.

Consequently, 47 patients were included in this study (67 [53–80] years, 34 males). Cardiovascular risk factors such as arterial hypertension, diabetes mellites or dyslipidemia were present in most patients. The predominant underlying disease was atherosclerosis (*n* = 33, 70.2%). In terms of vascular localization, strokes occurred more frequently in the anterior circulation (28 events, 59.6% vs. 19 events, 40.4%). Detailed information about patient and stroke characteristics are available in Table 2. DUS results were available in 37 patients (78.7%).

**Table 2:** Patient and stroke characteristics

Age (median, IQR)	67[53–80]	
NIHSS upon admission (median, IQR)	3[2–6]	
mRS at discharge (median, IQR)	2[1–3]	
–	*n*	%
*Gender*		
Female	13	27.7
Male	34	72.3
*Vascular territory*		
Anterior circulation	28	59.6
Posterior circulation	19	40.4
*Bleeding*		
Intracranial	1	2.2
Extracranial	0	0
*Risk factors*		
Hypertension	33	70.2
Diabetes mellitus	11	23.4
Dyslipidemia	18	38.3
Smoking	5	10.6
Previous stroke/TIA	18	38.3
Atrial fibrillation	8	17.0
*Underlying disease*		
Atherosclerosis	33	70.2
Cardiac	16	34.0
Dissection	2	4.3

*IQR* interquartile range, *mRS* modified Rankin Scale, *NIHSS* National Institutes of Health Stroke Scale, *TIA* transient ischemic attack

### Assessment of Extracranial ICA Stenosis

Pooled for both readers, a total of 20 stenoses (21.3%) in 17 patients (36.2%) were identified in CE-MRA of which 9 stenoses (9.6%) in 8 patients (17.0%) were classified as relevant (≥ 50% lumen reduction). REACT achieved a sensitivity of 95.0% and a specificity of 97.3% for all stenoses. For clinically relevant stenoses, REACT achieved a sensitivity of 88.9% and a specificity of 97.6%, meaning that 8 out of 9 relevant stenoses were correctly classified as relevant, while one stenosis was incorrectly classified as not relevant in the REACT. Diagnostic confidence was high in both sequences (both: 3, [3-3]; *p* = 0.22).

The interobserver agreement was almost perfect for both CE-MRA and REACT with κ = 0.87 and κ = 0.88, respectively. In addition, REACT achieved substantial agreement with CE-MRA in terms of disease grade κ = 0.83.

A detailed overview of the specific degrees of stenosis in CE-MRA, REACT and DUS is provided in the supplementary Table 1. Moreover, the supplementary data includes the confusion matrices, which encompass sensitivity and specificity for REACT with CE-MRA as the reference standard, as well as for REACT and CE-MRA with DUS results as the reference standard.

### Subjective Image Quality

Each reader evaluated 94 datasets (47 each for CE-MRA and REACT).

Table 3 shows the results of the subjective image quality assessment pooled for both readers.

**Table 3:** Comparison of subjective image quality, image noise, and artifact scores pooled for both readers as well as their interobserver agreement

	CE-MRA		REACT		*p* value	*W*(*p* value)
Image quality	Median [IQR]	Score ≥ 4 (*n*, %)	Median [IQR]	Score ≥ 4 (*n*, %)		
Aortic arch	4[4–4.5]	73(77.6)	4[3–4]	56(59.6)	**0.002**	0.76(**<** **0.001**)
CCA	4[4–5]	79(84.0)	4[4–4.5]	83(88.3)	0.59	0.70(**0.006**)
ICA1	4[4–5]	83(88.3)	5[4.5–5]	89(94.7)	**0.008**	0.67(**0.02**)
ICA2	4.5[4–5]	86(91.5)	5[4–5]	79(84.0)	0.53	0.72(**0.004**)
V1	3[3–4]	40(42.6)	3[2.5–3]	17(18.1)	**<** **0.001**	0.82(**<** **0.001**)
V2	4[3–4.75]	61(64.9)	4[3–4]	53(56.4)	**0.045**	0.87(**<** **0.001**)
V3	4[3–4.25]	60(63.8)	3.5[3–4]	46(48.9)	**0.03**	0.84(**<** **0.001**)
All vessels	4[3.57–4.43]	482(73.3)	3.79[3.43–4.14]	423(64.3)	**0.03**	0.85(**<** **0.001**)
Noise	4[3–4]	63(67.0)	4.5[4–5]	89(94.7)	**<** **0.001**	0.73(**0.002**)
Artifacts	5[4–5]	84(89.4)	5[4–5]	77(81.9)	0.60	0.46(0.72)

Bold indicates statistical significance

*CE* contrast-enhanced, *CCA* common carotid artery, *ICA1* extracranial segment of the internal carotid artery, *ICA2* petrous segment of the internal carotid artery, *REACT* Relaxation-Enhanced Angiography without Contrast and Triggering, *V1–3* vertebral artery segments, *W* Kendall’s coefficient of concordance

In CE-MRA, combined for both readers, 2.0% (13/658) segments were rated as grade 1, 6.1% (40/658) as grade 2, 18.7% (123/658) as grade 3, 42.1% (277/658) as grade 4 and 31.2% (205/658) grade as 5, resulting in a median of grade 4 [3.57–4.43]. In REACT, combined for both readers, 2.9% (19/658) segments were rated as grade 1, 8.2% (54/658) as grade 2, 24.6% (162/658) as grade 3, 37.5% (247/658) as grade 4 and 26.7% (176/658) as grade 5, resulting in a median of grade 3.79 [3.43–4.14], *p* < 0.03.

CE-MRA reached higher scores for the aortic arch (CE-MRA 4, [4–4.5] vs. REACT 4, [3–4]; *p* = 0.002) and at the vertebral arteries (e.g. V1: 3, [3–4] vs. 3 [2.5–3]; *p* < 0.001), while REACT achieved better values for the extracranial ICA (4, [3–4] vs. 5 [4.5–5]; *p* = 0.008). This segment achieved the most frequent rating of good or excellent (89/94; 94.7%) in REACT compared to CE-MRA (83/94; 88.3%). For the CCA (4, [4–5] vs. 4 [4–4.5]; *p* = 0.59) and the petrous segment of the ICA (4.5, [4–5] vs. 5, [4–5]; *p* = 0.53), there were no significant differences between both techniques.

In both sequences, image quality was not significantly affected by artifacts (5, [4–5] vs. 5, [4–5]; *p* = 0.60), while image noise was more pronounced in CE-MRA than in REACT (4, [3–4] vs. 4.5, [4–5]; *p* < 0.001).

Overall, the concordance between both readers was substantial to almost perfect with a Kendall’s coefficient in the range of *W* = 0.67 (extracranial ICA) to *W* = 0.87 (V3 segment), only artifact ratings showed a moderate agreement with *W* = 0.46. Kendall’s coefficients for all ratings are provided in Table 3.

### Fat-water Swapping Artifacts

No fat-water swapping artifacts were detected in REACT.

### Objective Image Quality

REACT achieved significantly higher aSNR and aCNR values than CE-MRA for both CCA and the extracranial ICA as well as for both vessel segments combined. Corresponding results are displayed in Table 4.

**Table 4:** Comparison of objective image quality

	CE-MRA	REACT	*p*
*aSNR (mean* *±* *SD)*			
CCA	16.3 ± 6.4	26.8 ± 14.2	**<** **0.001**
Extracranial ICA	20.2 ± 8.6	41.3 ± 20.9	**<** **0.001**
Combined	18.3 ± 6.7	34.1 ± 15.1	**<** **0.001**
*aCNR (mean* *±* *SD)*			
CCA	11.6 ± 4.9	21.6 ± 12.1	**<** **0.001**
Extracranial ICA	14.0 ± 7.0	33.6 ± 17.9	**<** **0.001**
Combined	12.8 ± 5.4	27.6 ± 12.7	**<** **0.001**

*aCNR* apparent contrast-to-noise ratio, *aSNR* apparent signal-to-noise ratio, *CE-MRA* contrast-enhanced magnetic resonance angiography, *CCA* common carotid artery, *ICA* internal carotid artery, *REACT* Relaxation-Enhanced Angiography without Contrast and Triggering, *SD* standard deviation

**Fig. 2:**
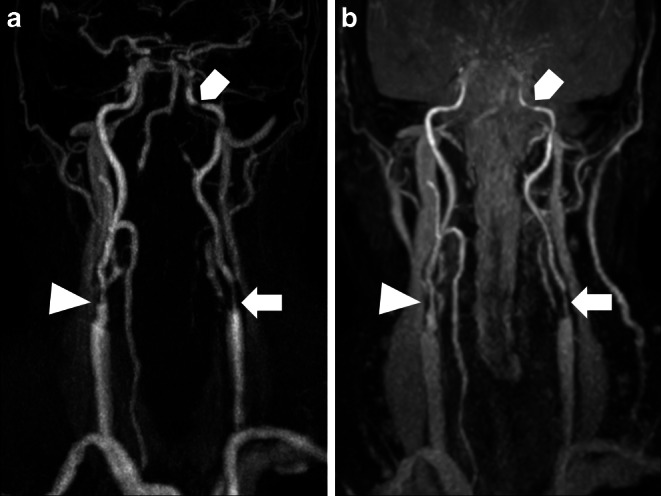
Maximum intensity projections of cervical arteries in coronal plane (slice thickness: 40 mm) in an 84-year-old male patient with acute embolic ischemia of the left anterior circulation with unknown onset. High grade stenosis of the proximal left extracranial (arrow w/head) and petrous (arrow w/o head) segment of the internal carotid artery (ICA) and serial middle grade stenosis in the right proximal ICA (arrowhead) are depicted equally in contrast-enhanced MRA (**a**) and Relaxation-Enhanced Angiography without Contrast and Triggering (REACT, water-only (**b**)). Contrast of the veins in contrast-enhanced MRA illustrates the potential venous contamination due to mistiming. Venous contamination is observed to a lesser extent in REACT, with visualization of the jugular vein, inferior petrosal, and sigmoid sinus

**Fig. 3:**
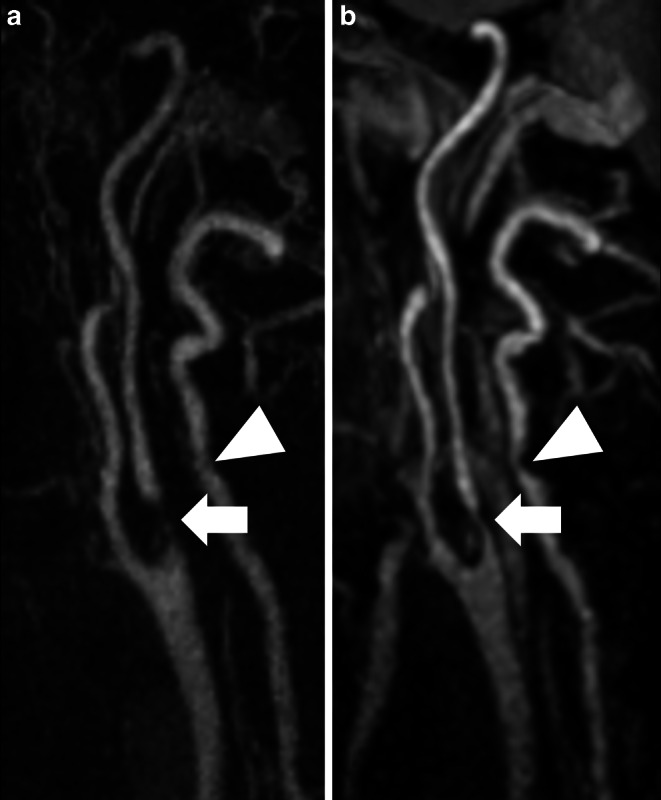
Maximum intensity projections of the right cervical arteries in sagittal plane (slice thickness: 20 mm) in a 92-year-old male patient presenting with acute embolic ischemia of the right hemisphere affecting the basal ganglia. High grade stenosis of the proximal extracranial grade internal carotid artery (ICA; arrow) and low grade stenosis of the vertebral artery V2 segment (arrowhead) are present in contrast-enhanced MRA (**a**) and Relaxation-Enhanced Angiography without Contrast and Triggering (REACT, water-only; (**b**)). REACT provides better delineation of high-grade ICA stenosis, whereas in contrast-enhanced MRA the noise predominates

**Fig. 4:**
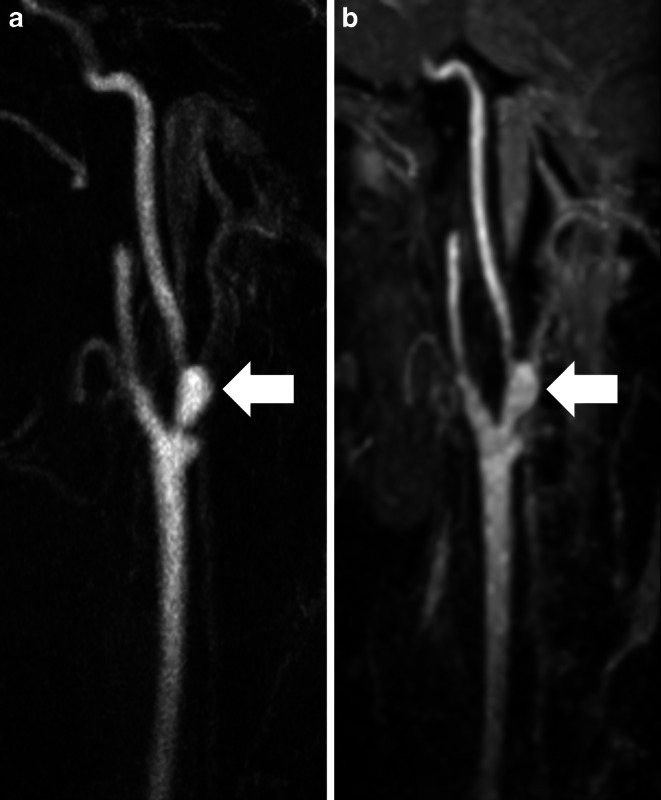
Maximum intensity projections of the right cervical arteries in sagittal plane (slice thickness: 30 mm) in a 41-year-old male patient presenting acute onset of paresthesia in the left hand. Pseudoaneurysm of the proximal extracranial internal carotid artery (arrow) with about 8 mm diameter is shown as additional finding in contrast-enhanced MRA (**a**) and Relaxation-Enhanced Angiography without Contrast and Triggering (REACT, water-only; (**b**)). Diagnosis was confirmed using duplex ultrasound. REACT provides better vessel delineation with less image noise

**Fig. 5:**
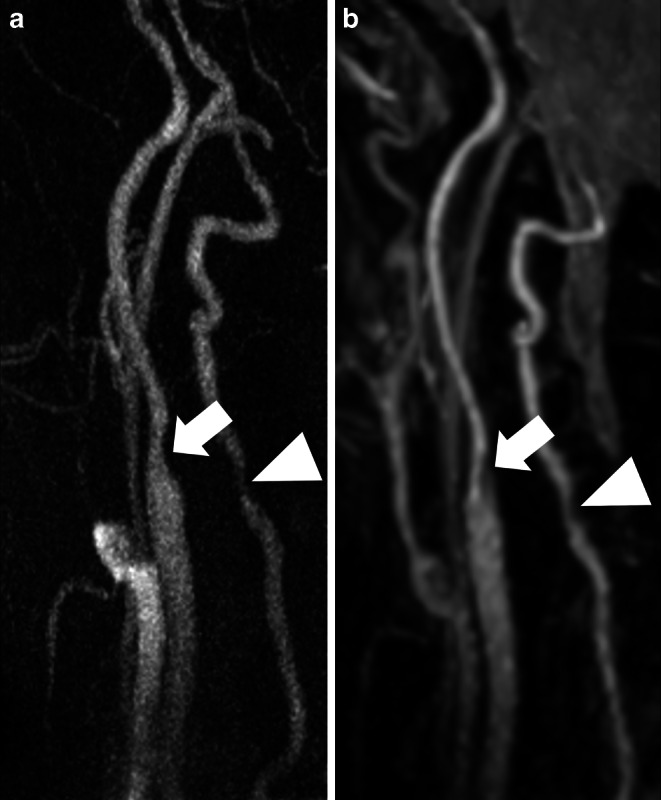
Maximum intensity projections of the left cervical arteries in sagittal plane (slice thickness: 30 mm) show a 80-year-old male patient with several border zone infarctions in the right anterior anterior circulation. Low grade stenosis of the proximal extracranial internal carotid artery (arrow) and low grade stenosis of the vertebral artery V2 segment (arrow head) are equally detectable in contrast-enhanced MRA (**a**) and Relaxation-Enhanced Angiography without Contrast and Triggering (REACT, water-only; (**b**)). Note the hyperintense signal of the adjacent plaque in REACT

Figure 2, 3, 4 and 5 show illustrative examples of low- and high-grade stenoses as well as additional vascular findings in REACT and CE-MRA.

## Discussion

In this retrospective, single-center study, the applicability of a novel 3D isotropic flow-independent non-CE-MRA technique (REACT) was evaluated for imaging of the extracranial arteries in AIS at 1.5 T by comparing objective and subjective image quality parameters and the detectability of ICA stenosis to CE-MRA.

The major findings are the following: In about three minutes, REACT obtained a high diagnostic performance regarding ICA stenosis. Furthermore, subjective image quality was comparable to CE-MRA with slightly inferior results at the aortic arch and vertebral arteries while REACT provided superior depiction of the extracranial ICA. In addition, the subjectively assessed effects of artefacts were equal in both techniques, whereas image noise yielded a higher impact in CE-MRA, which was confirmed in the objective evaluation of image quality with REACT showing higher aSNR and aCNR.

REACT yielded a high sensitivity (95%) and specificity (97%) for the detection of ICA stenosis. These findings are in line with previous studies at 3 T (reporting sensitivities of 90–94% and specificities of 95–100% [28, 29]) as well as to QISS-MRA at 1.5 T (sensitivity: 86%, specificity: 90% [30]). Furthermore, just as in the previous study at 3 T [29], neither the REACT nor the CE-MRA showed relevant impairment of diagnostic confidence. Intersequence agreement regarding stenosis grade of REACT was rated almost perfect in the current study (κ = 0.83), hence being comparable to previous results at 3 T (κ = 0.90, and κ = 0.89 respectively) [28, 29]. Stenosis agreement between QISS-MRA and CE-MRA at 1.5 T was also very high (r = 0.92) [30], but methodological differences hamper a direct comparison between correlation coefficients.

In line with QISS-MRA at 1.5 T [30] and 3 T [25, 26] as well as with REACT at 3 T [28, 29], the overall image quality of the non-CE-MRA technique in the present study was rated comparable but slightly inferior to CE-MRA. This could be explained by the inferior voxel size of REACT (1.5 mm^3^ compared to 0.7 mm^3^) which might be mitigated in the future using deep learning-based image reconstruction to improve spatial resolution [40]. Additionally, REACT provides a simultaneous readout of arteries and veins, which is beneficial in cardiothoracic applications [31, 33] but disadvantageous at the neck given the venous contamination of the small vertebral vessels [24, 31]. Adjustment of the T2 preparation pulse may improve the difference in contrast between arteries and veins, focusing on the divergent oxygenation of venous and arterial blood [24]. Like at 3 T, REACT provided equal (CCA and petrous segment) and superior results (extracranial ICA) for the carotid arteries compared to CE-MRA given its higher contrast and signal to surrounding tissue, while QISS-MRA showed inferior values than CE-MRA for this vessel segment [28, 30]. These findings underline the potential of REACT for carotid artery imaging and are consistent with significantly higher aSNR and aCNR at both CCA and ICA. These differences are due to distinct sequence parameters, primarily the higher acquired voxel size of REACT and even more pronounced than in previous studies at 3 T, where only values at the CCA were significantly higher [28]. Given the decrease in field strength, overall aSNR and aCNR values were lower at 1.5 T than at 3 T (e.g., mean aCNR of 33.6 at the ICA for REACT compared to 63.0 at 3 T) [15]. Fat-water swapping artifacts due to B0 inhomogeneities represent a common artifact of the mDIXON XD readout and were observed in 28.6–35.5% of patients in REACT studies of the cervical arteries at 3 T, affecting the left CCA and the left subclavian artery [28, 29, 39]. Interestingly, no fat-water swapping artifacts were observed in this study, mostly due to lower B0 inhomogeneities at 1.5 T [41].

QISS-MRA and TOF-MRA are the most frequently used non-CE-MRA techniques for imaging of the supraaortal arteries at 1.5 T and enable the depiction of the intracranial arteries, whereas the REACT sequence is unable to delineate the intracranial arteries given its T2 preparation pulse. However, both QISS- and TOF-MRA require long acquisition times of up to seven respective nine minutes at 1.5 T, whereas REACT takes about three minutes [30, 42]. While even shorter acquisition times have already been reported for 2D QISS-MRA at 3 T using deep neural network-based image processing, unknown reconstruction times and the required additional central processing unit cluster limits its feasibility for other centers [14]. The recently introduced two-point Dixon spoiled gradient-echo FLEXA technique uses parallel imaging and compressed sensing for acceleration of data acquisition enabling non-CE-MRA of the cervical arteries at 3 T in 1:28 min [29]. However, its direct comparison to CE-MRA and general applicability at 1.5 T is unknown.

While REACT is a flow-independent technique based on the specific relaxation properties of blood [24], both TOF- and QISS-MRA are flow-dependent techniques being prone to flow artifacts, leading to overestimation for ICA stenosis in TOF-MRA due to signal intensity saturation of blood flow distal to high-grade stenosis [25, 26, 30]. While QISS-MRA, being a 2D sequence, provides a high in-plane resolution (0.5 × 0.5 mm) compared to REACT (1.5 × 1.5 mm), the mDIXON readout of the latter enables the acquisition of 3D isotropic datasets with a higher through-plane resolution (1.5 vs. 2 mm), allowing post-acquisition angulation and reformatting in any arbitrary orientation, which can be advantageous for stenosis assessment [30]. While QISS-MRA provides best image quality when employing cardiac gating [26] and uses an image-based navigator to reduce swallowing artifacts at 1.5 T [30], REACT can be acquired without any triggering which is beneficial in daily clinical practice given that stroke imaging is generally performed without pulse or cardiac synchronization [24].

There are some limitations to this study: First, REACT was only compared with the MRI reference standard (CE-MRA) and neither with DSA nor other non-CE-MRA techniques such as TOF-MRA. As for QISS-MRA at 1.5 T [30], the current study used subtraction images of CE-MRA for intraindividual comparison, hampering the direct comparison to previous studies at 3 T [28, 29]. Second, the sample size was limited to 47 patients, which may not be sufficient to draw definitive conclusions. However, this sample size was larger than in comparable studies, e.g., Peters et al. (31 patients) or Pennig et al. (35 patients) [28, 30]. Third, given the clear visual distinction of the two sequences, it was not possible to blind the readers to the sequence technique. Fourth, no core lab-adjudicated images were used instead subjective results were provided by readers with limited experience in MRA (two and six years, respectively). To mitigate the potential for misjudgment, readers were instructed by an expert prior to evaluation and the mean value of both readers was calculated and used for further analysis. Fifth, the results were augmented by supplementary DUS findings from the medical reports. However, these should be considered with caution, as the data were incomplete, the examinations were performed with awareness of the MRI findings, and no standardized protocol was provided. Sixth, due to the small sample size, no assessment of other causes for AIS, e.g., atheromatous plaque or dissection, was conducted in this study, which could nurture future research. Of note, Hoyer et al. demonstrated the performance of REACT for plaque depiction at 3 T [29]. Seventh, the use of SNR and CNR values as an objective measure of image quality is limited for acceleration techniques such as parallel imaging and compressed sensing [43, 44]. However, the results in this study are consistent with the subjective image impression and in line with previously reported results at 3 T [28]. Lastly, the compressed SENSE acceleration factor was based on clinical experience rather than profound investigation. Higher acceleration factors are likely possible, especially with AI-based reconstruction, which could enhance REACT’s clinical use as an alternative to CE-MRA [27].

## Conclusions

REACT enables non-contrast-enhanced visualization of the cervical arteries in a short scan time at 1.5 T while achieving an image quality comparable to CE-MRA which may allow accurate assessment of extracranial ICA stenosis in AIS. Therefore, its clinical application can be expanded to lower field strengths.

## Supplementary Information

The supplementary information provides a detailed overview of the ACI stenosis gradings in CE-MRA, REACT, and the additional DUS reports, accompanied by an analysis of the respective diagnostic accuracies.

## Abbreviations

aCNR: Apparent contrast-to-noise ratio
AIS: Acute ischemic stroke
aSNR: Apparent signal-to-noise ratio
CCA: Common carotid artery
CE-MRA: Contrast-enhanced magnetic resonance angiography
DSA: Digital subtraction angiography
DUS: Duplex ultrasound
ICA: Internal carotid artery
mRS: Modified Rankin Scale
NIHSS: National Institutes of Health Stroke Scale
Non-CE-MRA: Non-contrast-enhanced magnetic resonance angiography
QISS: Quiescent interval slice-selective
REACT: Relaxation-Enhanced Angiography without Contrast and Triggering
TOF: Time-of-flight
