# Supplementary material for: Non-contrast-enhanced MR-angiography of Extracranial Arteries in Acute Ischemic Stroke at 1.5 Tesla Using Relaxation-Enhanced Angiography Without Contrast and Triggering (REACT)
Source: Clin Neuroradiol. 2024 Sep 24;35(1):105–14. doi: 10.1007/s00062-024-01458-4 (PMC11832615; doi:10.1007/s00062-024-01458-4)
Supplement: Supplementary file 1 — The supplementary information provides a detailed overview of the ACI stenosis gradings in CE-MRA, REACT, and the additional DUS reports, accompanied by an analysis of the respective diagnostic accuracies. [file 62_2024_1458_MOESM1_ESM.pdf]

Supplementary data

Clinical Neuroradiology

Non-contrast-enhanced MR-angiography of Extracranial Arteries in Acute Ischemic Stroke at 1.5 Tesla using Relaxation-Enhanced Angiography without Contrast and Triggering (REACT)

Jan P Janssen[1]*; Sarah Rose[1]; Kenan Kaya[1]; Robert Terzis[1]; Robert Hahnfeldt[1]; Roman J Gertz[1]; Lukas Goertz[1]; Andra-Iza Iuga[1]; Jan-Peter Grunz[2, 3]; Christoph Kabbasch[1]; Philip Rauen[1]; Thorsten Persigehl[1]; Kilian Weiss[4]; Jan Borggrefe[5]; Lenhard Pennig[1]; Carsten Gietzen[1]

Affiliations:

[1]Institute for Diagnostic and Interventional Radiology, Faculty of Medicine and University Hospital Cologne, University of Cologne, Cologne, Germany

[2]Institute for Diagnostic and Interventional Radiology, University Hospital Wuerzburg, Wuerzburg, Germany

[3]University of Wisconsin–Madison, Madison, WI, United States

[4]Philips GmbH, Hamburg, Germany

[5]Department of Radiology, Neuroradiology and Nuclear Medicine, Johannes Wesling University Hospital, Ruhr University Bochum, Bochum, Germany

(*)Correspondence:
Jan Paul Janssen
Institute for Diagnostic and Interventional Radiology, Faculty of Medicine and University Hospital Cologne, University of Cologne, Kerpener Straße 62, 50937 Cologne, Germany

Tel: +49/22147882035
jan.janssen@uk-koeln.de

Table 1 Stenosis grading including duplex ultrasound data from medical records

	**No stenosis**	**<50%**	**50-69%**	**70-99%**	**Total occlusion**
CE-MRA (94 ICAs in 47 patients)	74 (78.7%)	11 (11.70%)	2 (2.1%)	5 (5.3%)	2 (2.1%)
REACT (94 ICAs in 47 patients)	73 (77.7%)	12 (12.8%)	5.5 (5.9%)	3.5 (3.7%)	1 (1.1%)
DUS (74 ICAs in 37 patients)	54 (73.0%)	14 (18.9%)	3 (4.1%)	3 (4.1%)	1 (1.4%)

CE, contrast-enhanced; DUS, duplex ultrasound; ICA, internal carotid artery; REACT, Relaxation-Enhanced Angiography without Contrast and Triggering

Confusion matrix 1 Index test = REACT; Reference standard = CE-MRA; all stenosis

All stenosis	CE-MRA stenosis (20)	CE-MRA no stenosis (74)
REACT stenosis (21)	19	2
REACT no stenosis (73)	1	72

Sensitivity = 95.0%; Specificity = 97.3%

CE, contrast-enhanced; REACT, Relaxation-Enhanced Angiography without Contrast and Triggering

Confusion matrix 2 Index test = REACT; Reference standard = CE-MRA; relevant stenosis

Relevant stenosis (≥ 50%)	CE-MRA stenosis (9)	CE-MRA no stenosis (85)
REACT stenosis (10)	8	2
REACT no stenosis (84)	1	83

Sensitivity = 88.9%; Specificity = 97.6%

CE, contrast-enhanced; REACT, Relaxation-Enhanced Angiography without Contrast and Triggering

Confusion matrix 3 Index test = REACT; Reference standard = DUS; all stenosis

All stenosis	DUS stenosis (21)	DUS no stenosis (53)
REACT stenosis (19)	15	4
REACT no stenosis (55)	6	49

Sensitivity = 71.4%; Specificity = 92.5%

DUS, duplex ultrasound; REACT, Relaxation-Enhanced Angiography without Contrast and Triggering

Confusion matrix 4 Index test = REACT; Reference standard = DUS; relevant stenosis

Relevant stenosis (≥50%)	DUS stenosis (7)	DUS no stenosis (67)
REACT stenosis (8)	4	4
REACT no stenosis (66)	3	63

Sensitivity = 57.1%; Specificity = 94.0%

DUS, duplex ultrasound; REACT, Relaxation-Enhanced Angiography without Contrast and Triggering

Confusion matrix 5 Index test = CE-MRA; Reference standard = DUS; all stenosis

All stenosis	DUS stenosis (21)	DUS no stenosis (53)
CE-MRA stenosis (18.5)	15.5	3
CE-MRA no stenosis (55.5)	5.5	50

Sensitivity = 73.8%; Specificity = 94.3%

CE, contrast-enhanced; DUS, duplex ultrasound

Confusion matrix 6 Index test = CE-MRA; Reference standard = DUS; relevant stenosis

Relevant stenosis (≥50%)	DUS stenosis (7)	DUS no stenosis (67)
CE-MRA stenosis (8)	5	3
CE-MRA no stenosis (66)	2	64

Sensitivity = 71.4%; Specificity = 95.5%

CE, contrast-enhanced; DUS, duplex ultrasound
